# Analysis of the prognostic, diagnostic and immunological role of HSP90α in malignant tumors

**DOI:** 10.3389/fonc.2022.963719

**Published:** 2022-09-08

**Authors:** Zhimin Yuan, Longhao Wang, Cheng Chen

**Affiliations:** ^1^ Xi’an Jiaotong University, Xi’an, China; ^2^ Department of Clinical Laboratory, Shaanxi Provincial Cancer Hospital, Xi’an, China; ^3^ Department of Otorhinolaryngology-Head and Neck Surgery, Shanghai Ninth People’s Hospital, Shanghai Jiaotong University, Shanghai, China; ^4^ Department of General Dentistry/Key Laboratory of Shaanxi Province for Craniofacial Precision Medicine Research, College of Stomatology, Xi’an Jiaotong University, Xi’an, China

**Keywords:** diagnostic, prognostic, pan-cancer, HSP90α, HSP90AA1

## Abstract

Heat shock protein 90α (HSP90α) encoded by the HSP90AA1 gene, is the stress inducible isoform of the molecular chaperone HSP90, and was demonstrated as a promising hallmark to diagnose, prognosis in malignant tumors. This study is to evaluate the value of HSP90α in diagnosis, prognosis and immunotherapy of malignant tumors by investigating the expression of HSP90α in plasma of various tumors and analyzing the expression of HSP90α at gene and protein levels *via* pan-cancer database. We founded that levels of HSP90α in malignant tumors groups were significantly higher than healthy controls in serum. Pan-cancer analysis showed that HSP90AA1 was highly expressed in 27 of 33 tumors, but low in individual cancers (such as renal malignancies). The plasma HSP90α level was positively correlated with the stage of malignant tumor, but there was no significant difference between HSP90AA1 and the stage of most tumors. Cox regression analysis showed that HSP90AA1 expression was significantly correlated with OS in only 6 of the 32 cancers, including LIHC, KIRC, HNSC, LUAD, BRCA and MESO. Up-regulation of HSP90AA1 in most tumors was positively correlated with PDCD1LG2 and CD274 immune checkpoint genes. T cell CD8+ was positively correlated with HSP90AA1 in COAD, DLBC and UVM, and negatively correlated with HSP90AA1 in ESCA, GBM, HNSC, KIRC, KIRP, UCEC and STAD. The AUC of HSP90α are generally high in different tumor groups, which indicated its diagnostic value in malignant tumors. In conclusion, serum HSP90α in patients with malignant tumor is generally elevated, which is of positive significance as an independent diagnosis and combined diagnosis. However, we found that the expression level of HSP90AA1 gene in most tumors was not completely consistent with the serum level, and even down-regulated in some tumors. Plasma levels can be used as biomarkers of poor prognosis in some tumors, but it cannot be used as a biomarker for poor prognosis of all tumors, and more in-depth studies are needed.

## Introduction

Cancer is a major health issue globally, which is responsible for an estimated of 10 million deaths each year (1). Though cancer has been studied for decades, it is continuously a leading cause of death. At present, cancer treatment methods mainly include surgery, chemotherapy, radiotherapy, targeted therapy and immunotherapy. Although these therapies have achieved some clinical success, the prognosis and survival rate of patients are still not satisfactory due to drug resistance and side effects (2, 3). Therefore, it is imperative to find therapeutic targets and potential tumor-sensitive biomarkers for early diagnosis, clinical cure and survival improvement (4).

Tumorigenesis is a multi-step, multi-layered process, including oncogene activation, suppression of tumor suppressor genes, genomic instability, epigenetic modification and abnormal cell signaling, resulting in the production of abnormal proteins and stress signals (5, 6). Add the formation of tumor microenvironment also provides a stressful living environment for the development of malignant tumor cells, such as hypoxia (7), malnutrition, ATP depletion and long-term chronic inflammation (8, 9). Under this prolonged stress, cells react to produce heat shock proteins (HSPs), as molecular chaperones involved in protein synthesis, folding, assembly, transportation and degradation, have a variety of biological functions. Many clinical studies have found that heat shock proteins (HSPs) are a promising tumor marker, which are highly expressed in various types of cancers and many middle-to-late tumor tissues, and it is associated with poor prognosis in cancer patients (10–12).

Among HSPs, heat shock protein 90 (HSP90) is highly conserved and mainly helps client protein to mature (13). Clinical studies have found that one of the four members of HSP90, HSP90α, is overexpressed in the serum or tissues of many patients with malignant tumors, such as lung cancer (14), prostate cancer (15), pancreatic ductal adenocarcinoma (16), liver cancer (17, 18) and pancreatic cancer (19), etc. Moreover, correlation analysis of some tumor-related clinical studies found that HSP90α was positively related to the metastasis and malignancy (20), and it can be used as a biological marker for diagnosis of tumors (21, 22). In all, the abnormal expression of HSP90α in many malignancies has increased in recent studies, and the authors felt it necessary to evaluate its clinical value and expression level in different malignancies as a whole.

HSP90α encoded by the HSP90AA1 gene, which encodes two different mRNA transcripts at different transcription start sites. There are some heat shock elements (HSE’s) upstream of HSP90α, which bind to HSF1 to induce expression of HSP90α (23). In addition to the main transcription factor HSF1, transcription of HSP90AA1 is regulated by a variety of other transcription complexes (24). The proto-oncogene MYC also induces the expression of HSP90AA1, and the decreased expression of HSP90α suggests that HSP90AA1 is necessary for MYC to drive transformation (25). In biology, HSP90α is currently thought to play a role of secretory extracellular factors in wound healing and inflammation in addition to its intracellular role. These two processes are often hijacked by cancer, resulting in malignant cell motility, metastasis and extravasation (26). This also briefly explains the elevated plasma HSP90α levels in patients with advanced malignant tumors and its potential as a tumor biomarker. However, most studies of HSP90α have involved specific types of cancer. Therefore, in-depth research on the regulatory function and molecular mechanism of HSP90α in pan-cancer data sets can provide new directions and strategies for clinical treatment of cancer. Moreover, by analyzing the expression of HSP90α in serum through generalized cancer, it can provide more comprehensive data support for biomarkers in clinical diagnosis of cancer.

This study systematically described the expression and prognostic value of HSP90α in pan-cancer, and evaluated its diagnostic value in pan-cancer. We combined serological laboratory data in our hospital with TCGA database carcinoma analysis to investigate the roles of HSP90α in prognosis, diagnosis and immune response, etc. We found that the high expression of HSP90α significantly correlated with a poor prognosis of several types of cancer. Upregulation of HSP90AA1 was associated with the increased CD8+ T cells. The HSP90AA1 expression exhibited strong correlations with immune checkpoint genes according to pan-cancer analysis. Our pan-carcinomatous analysis and clinical data analysis provide a deeper understanding of the differences in HSP90AA1 gene and HSP90α protein expression in plasma and tissues of different cancers and identify strategies that may be used to promote collaborative activity in prognosis, diagnosis, and immunotherapy.

## Materials and methods

### HSP90α-related clinical case data collection

The population treated in Shaanxi Cancer Hospital from 2017 to 2021, including cancer patients and normal physical examination individuals, were collected. The cancer group with HSP90α index detected was screened. The inclusion criteria are: 1) patients were assessed HSP90α before and after operation, radiation therapy and/or chemotherapy. 2) The values of HSP90α were evaluated at first routine examine of patients. The cancer group in this study were patients with a single tumor, and patients with other diseases, such as infection, inflammation and other diseases under stress, were excluded. In addition, the control group tested for HSP90α was screened out, and the inclusion criteria: 1) Adults who report the healthy results of a medical examination. 2) Individuals were not diagnosed with any neoplastic diseases. Basic clinic-pathological data of the enrolled population, such as gender, age, tumor type, TNM classification and metastasis were collected. The staging of malignant tumors was classified according to American Joint Committee on Cancer classification (AJCC 7th edition, 2017). All patients’ identity remained anonymous, and the requirement for informed consent was waived due to the observational nature of the study, as reported elsewhere. This study was approved by the Ethics Committee of Shaanxi Provincial Cancer Hospital (No. 2021089).

### Detection and evaluation of HSP90α in plasma of cancer patients

The levels of plasma HSP90α were measured by ELISA kit (Yantai Protgen Biotechnology Development Co., Ltd., Yantai, China). The medical device registration certificate and product technical requirements number is 20173400448. The detection principle is the double-antibody sandwich method, precoated with HSP90α monoclonal antibody (E7) in the microplate, labeled with HSP90α monoclonal antibody (F6) in horseradish peroxidase, to form the antibody-antigen-enzyme-conjugated antibody complex. The detection of HSP90α was performed according to the manufacture’s instruction. The protocol of HSP90α evaluation was followed by manufacture’s instruction. We collected 2 mL blood samples with EDTA-K2 anticoagulant from patients and controls. The kits were preincubated at 37°C for 30 min before tests. The collected samples were centrifuged (3000 rpm, 10 min); and the blood samples were diluted solution with removed plasmas; then after loading the standards, all samples were added into 96-well plates, 50 μL of each, with addition 50μL of anti- HSP90αHRP-conjugated antibody. After one-hour incubation, they were gentle shaken. After six times washes, 50μL peroxide and 50μL 3, 3′, 5, 5′ - tetramethylbenzidine were added for 20 min chromogenic reaction at 37°C, then terminating reaction by acid stop buffer. Finally, we measure the OD values at the wavelength of 450/620 nm (450 nm for detection, while 620 nm as reference). We calculated the concentration of HSP90α through a standard curve of optical density values.

### Expression pattern, distribution and clinicopathological correlation analysis of HSP90AA1 in human pan-cancer

The dysregulation of HSP90AA1 expression in different cancer types and normal tissues was studied by downloading the data for from The Cancer Genome Atlas (TCGA) database (https://genome-cancer.ucsc.edu) and GTEx database for normal tissues (27–29). RNA sequencing data and clinical follow-up information for cancer patients with 33 types of cancers, including adrenocortical carcinoma(ACC), bladder urothelial carcinoma (BLCA), breast invasive carcinoma (BRCA), cervical squamous cell carcinoma (CESC), cholangiocarcinoma (CHOL), colon adenocarcinoma (COAD), lymphoid neoplasm diffuse large B cell lymphoma (DLBC), esophageal carcinoma (ESCA), glioblastoma(GBM), brain lower grade glioma (LGG), head and neck squamous cell carcinoma (HNSC), kidney chromophobe (KICH), kidney renal clear cell carcinoma (KIRC), kidney renal papillary cell carcinoma (KIRP), acute myeloid leukemia (LAML), liver hepatocellular carcinoma (LIHC), lung adenocarcinoma (LUAD), lung squamous cell carcinoma(LUSC), mesothelioma (MESO), ovarian serous cystadenocarcinoma (OV), pancreatic adenocarcinoma (PAAD), pheochromocytoma and paraganglioma (PCPG), prostate adenocarcinoma (PRAD), rectum adenocarcinoma (READ), sarcoma (SARC), skin cutaneous melanoma (SKCM), stomach adenocarcinoma (STAD), testicular germ cell tumors (TGCT), thyroid carcinoma (THCA), thymoma (THYM), uterine corpus endometrial carcinoma (UCEC), uterine carcinosarcoma (UCS), and uveal melanoma (UVM), were obtained from the TCGA database. The abscissa represents different groups of samples, and the ordinate represents the expression distribution of the HSP90AA1 gene, different colors represent different groups, top-left represents the significance p-value, *p < 0.05, **p < 0.01, ***p < 0.001, asterisks (*) stand for significance levels. The statistical difference of two groups was compared through the Wilcox test. To investigate the relationship between HSP90AA1 expression and clinicopathological features of various cancers, we evaluated HSP90AA1 expression in patients with stage I, II, III, and IV cancers. The statistical difference of stages was compared through Kruskal-Wallis test and the Mann-Whitney U test was used for comparison between stages.

### Prognostic analysis

The connection between the HSP90AA1 expression and the prognosis of cancer patients, including overall survival (OS), progression free survival (PFS), disease free survival (DFS) and disease-specific survival (DSS) in 33 types of cancer in TCGA database was analyzed by Kaplan-Meier curves and forest plots. The hazard ratios (HRs) and 95% confidence intervals were calculated using univariate survival analysis. P value < 0.05 was considered as statistically significant. Log rank P value < 0.05 was considered as statistically significant.

### Correlation analysis between HSP90AA1 genes with pathway

RNA-sequencing expression (level 3) profiles and corresponding clinical information for LIHC and KIRC were downloaded from the TCGA dataset (https://portal.gdc.com). R software GSVA package was used to analyze, choosing parameter as method=‘ssgsea’. The correlation between genes and pathway scores was analyzed by Spearman correlation. All the analysis methods and R packages were implemented by R version 4.0.3. P value < 0.05 was considered statistically significant. The authors selected pathways that are relevant to the mechanisms of cancer research, PI3K/AKT/mTOR pathway, EMT marker gene, DNA damage repair, P53 signaling pathway, inflammatory features of tumor, tumor proliferation characteristics, MYC target genes, the inflammatory response and transforming growth factor beta (TGFB), respectively.

### Pan-cancer analysis of the relationship between the HSP90AA1 gene expression and TMB or MSI

TMB and MSI scores were taken from TCGA. Spearman method was used to analyze the correlation between HSP90AA1 expression and TMB or MSI. The horizontal axis in the figure represents the correlation coefficient between HSP90AA1 and TMB or MSI, the ordinate is different types of cancer, the size of the dots in the figure represents the size of the correlation coefficient, and the different colors represent the significance of the p value.

### Pan-cancer analysis of the correlation of the HSP90AA1 expression with tumor cell infiltration and immune modulator genes

The data from 33 types of cancer and normal tissues in TCGA were downloaded from the Genomic Data Common (GDC) portal. After these data were standardized, the differential expression of HSP90AA1 gene was analyzed by using R package limma. The cell line expression matrix of 32 types of tumors was obtained from the dataset of Cancer Cell Line Encyclopedia (CCLE) (https://portals.broadinstitute.org/ccle/about). The above analysis was constructed by the R v4.0.3 software package ggplot2 (v3.3.3).

To assess the reliable results of immune score evaluation, we used immuneeconv. It is an R software package that integrates six latest algorithms, including TIMER, xCell, MCP-counter, CIBERSORT, EPIC and quanTIseq. SIGLEC15, IDO1, CD274, HAVCR2, PDCD1, CTLA4, LAG3 and PDCD1LG2 are the transcripts which associated with the immune checkpoint. Extracting the expression of 8 genes, observing the expression value of the immune-checkpoint-related genes. All the analysis methods and R package were implemented by R version 4.0.3. If not stated otherwise, two-group data performed by wilcox test. *P* values less than 0.05 were considered statistically significant (*p  <  0.05).

### Correlation between HSP90AA1 and immune cells in multiple cancers

The HSP90AA1 expression profile and the abundance of immune infiltrates in pan-cancer were analyzed using six online databases, namely CIBERSORT, EPIC, MCPCOUNTER, QUANTISEQ, TIMER and XCELL. Spearman correlation heat map of immune score or immune checkpoint related genes and HSP90AA1 gene expression was established. The horizontal axis of the heat map represents different types of cancer, the vertical axis represents different immunity scores, and different colors represent correlation coefficients. Statistical analysis was performed using R software V4.0.3. R > 0.1 and p value < 0.05 were set as selection criteria for identifying as statistically significant.

### Evaluation and analysis of independent combined diagnostic efficacy of HSP90α

**Figure 1:**
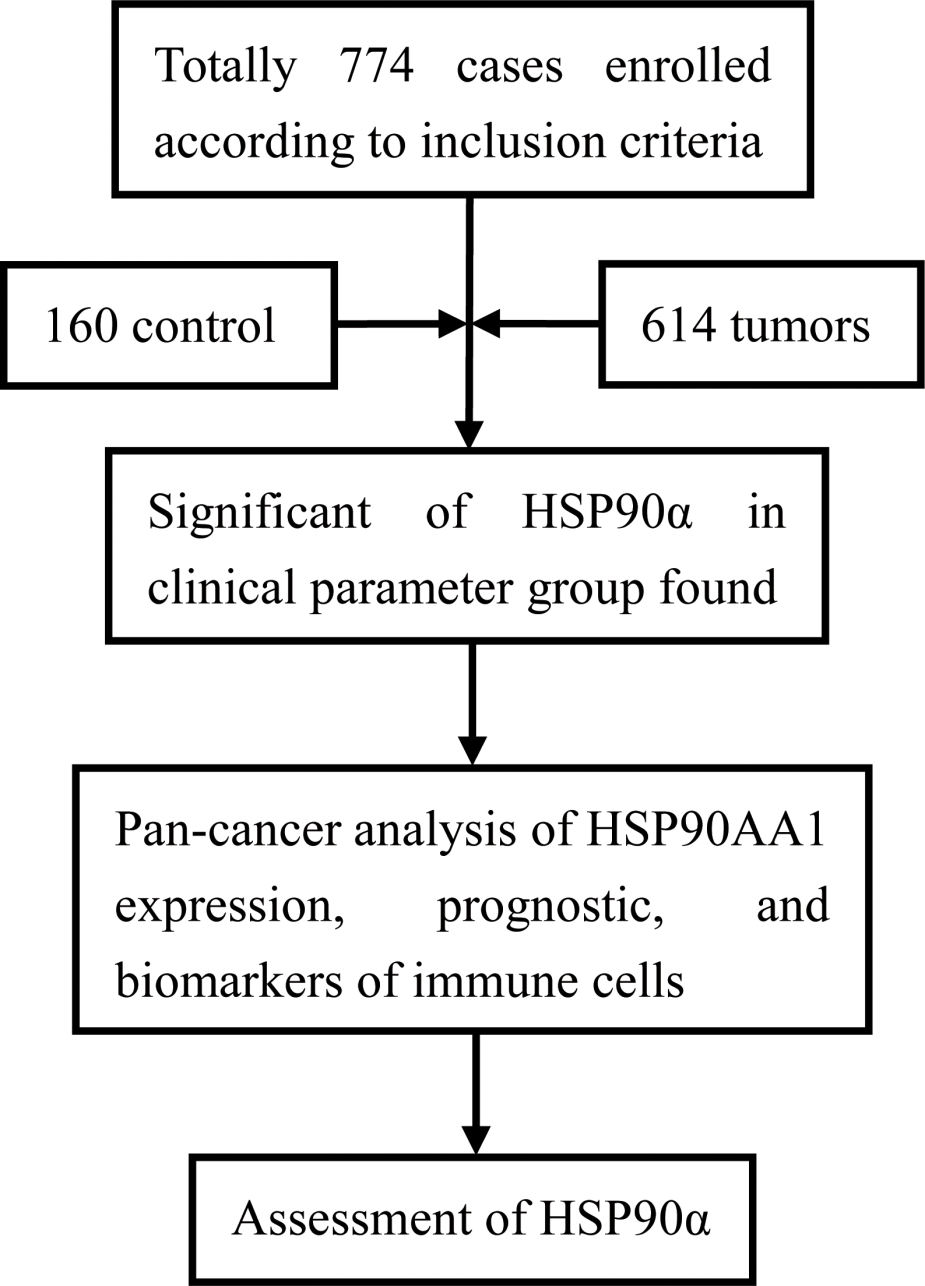
The current study was presented by the flowchart.

ROC curve was used to analyze and predict the diagnostic value of HSP90α in plasma, and the combined diagnostic efficacy was evaluated by combining with carcinoma embryonic antigen (CEA) and alpha-fetoprotein (AFP) tumor markers. HSP90α, CEA and AFP were three independent diagnostic groups, while HSP90α+AFP, HSP90α+CEA and HSP90α+CEA+AFP were three combined diagnostic groups. To further compare the predictive value of plasma HSP90α with other conventional tumor markers, correlation analysis was carried out between the plasma HSP90α and other clinical markers. All data were presented as the mean ± SD. The patients were analyzed as tumor and control groups. The Wilcoxon Mann-Whitney test and Kruskal-Wallis test were performed by SPSS 20.0 software (SPSS, Chicago, IL, USA) for comparison of HSP90α. P < 0.05 was considered as statistically significant. The paired comparison of ROC curves and the box plot of HSP90α expression in different cancers were plotted by GraphPad Prism 9.0 software (GraphPad Software, Inc., San Diego, CA, USA). The optimal cut-off point of HSP90α was determined by the proportion of sensitivity versus specificity of threshold values. The flowchart of this study was as follows (**Figure 1**).

## Results

### Description of clinical pathological data and HSP90α expression in Plasma

A total number of 774 cases were enrolled in this study, including 614 cases with malignant tumors and 160 healthy controls. Levels of HSP90α in malignant tumors groups were significantly higher than healthy controls (*p* < 0.001). The plasma levels of HSP90α positively correlated with malignancy cancer in staging (*p* < 0.001). No significant difference in HSP90α were observed in gender (*p* = 0.058), age (*p* = 0.665), classification (*p* = 0.610) and metastasis groups (*p* = 0.113). As shown in **Table 1**.

**Table 1:** Associations between HSP90α levels and clinical characteristics of malignant tumors.

Variables	Total (n=774)	HSP90α (ng/ml)	*p-*value
group malignant tumors	614	81.25 ± 43.16	<0.001*
healthy controls	160	38.04 ± 12.91	
age <60y	271	82.65 ± 44.61	0.665
≥60y	343	79.81 ± 41.69	
gender male	386	77.65 ± 40.05	0.058
Female	228	86.83 ± 47.09	
staging I	44	66.22 ± 32.75	<0.001*
II	109	72.16 ± 40.35	
III	204	76.94 ± 40.71	
IV	257	90.65 ± 45.47	
metastasis yes	185	85.38 ± 44.05	0.113
No	429	79.20 ± 42.06	
classification small-cell carcinoma	59	74.59 ± 35.80	0.610
Adenocarcinoma	423	82.26 ± 44.09	
squamous-cell carcinoma	132	80.10 ± 42.38	
radiation and/or chemotherapy before	614	81.25 ± 43.20	0.005*
After	614	74.31 ± 44.26	
CEA (ng/ml) <5.5	235	78.44 ± 42.40	0.131
≥5.5	379	82.69 ± 43.33	
AFP (IU/ml) <6.05	351	82.54 ± 44.74	0.773
≥6.05	263	79.09 ± 40.55	

*p < 0.05 was considered statistically significant. Carcinoma Embryonic Antigen (CEA), Alpha-Fetoprotein (AFP). CEA: 0-5.5 ng/ml, AFP: 0-6.05 IU/ml.

**Figure 2:**
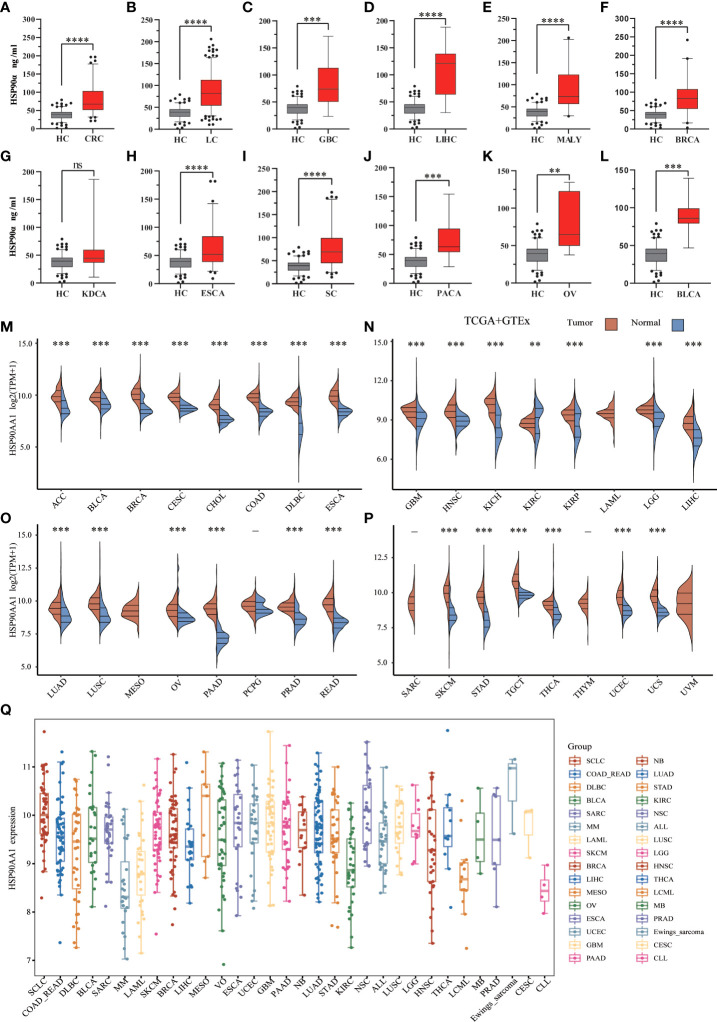
.Expression analysis for HSP90α and HSP90AA1 in multiple cancers. (**A–L**) The expression of plasma HSP90α level in tumor groups and healthy control; (**M–P**) The expression of HSP90AA1 gene in 33 types of human cancer tissues and normal tissues based on TCGA+GTEx; (**Q**) The expression distribution of HSP90AA1 gene in 32 types of human cancer tissues and normal tissues based on CCLE. **p < 0.01, ***p < 0.001, ****p < 0.0001, the asterisk represents the degree of importance (*p). The significance of the two groups of samples passed the Wilcox test. ns, no statistical significance.

There were 12 types of tumor groups, including Colorectal Cancer (CRC) (93), Lung Cancer (LC) (227), Gallbladder Cancer (GBC) (10), Liver Cancer (LIHC) (11), Malignant Lymphoma (MALY) (24), Breast Cancer (BRCA) (40), Kidney Cancer (KDCA) (8), Esophageal Cancer (ESCA) (77), Stomach Cancer (SC) (93), Pancreatic Cancer (PACA) (14), Ovarian Cancer (OV) (10), and Bladder Cancer (BLCA) (7). The statistical summary of HSP90α in different groups were showed in **Table 2**. The plasma HSP90α level were compared in different tumor groups with healthy control. As shown in **Figures 2A–L**.

**Table 2:** Levels of plasma of Hsp90α in various tumors and healthy control group.

Group	Cases (N)	HSP90α (ng/ml)	Z	*p-*value
HC	160	38.04 ± 12.91	/	/
CRC	93	80.10 ± 43.00	9.152	<0.0001***
LC	227	86.05 ± 41.18	13.18	<0.0001***
GBC	10	86.36 ± 48.69	4.055	0.0006***
LIHC	11	111.93 ± 51.81	5.612	<0.0001***
MALY	24	91.02 ± 50.50	6.320	<0.0001***
BRCA	40	86.50 ± 43.97	7.912	<0.0001***
KDCA	8	60.13 ± 53.25	1.478	0.107
ESCA	77	63.74 ± 35.52	5.702	<0.0001***
SC	93	78.06 ± 45.85	8.435	<0.0001***
PACA	14	74.20 ± 37.13	4.052	0.0006***
OVC	10	77.50 ± 39.40	3.663	0.003**
BLCA	7	89.52 ± 27.64	4.193	0.0003***

N, numbers of cases; Z, value of Z-test; p-value: **p < 0.01, ***p < 0.001. Healthy Control (HC), Colorectal Cancer (CRC), Lung Cancer (LC), Gallbladder Cancer (GBC), Liver Cancer (LIHC), Malignant Lymphoma (MALY), Breast Cancer (BRCA), Kidney Cancer (KDCA), Esophageal Cancer (ESCA), Stomach Cancer (SC), Pancreatic Cancer (PACA), Ovarian Cancer (OVC), Bladder Cancer (BLCA).

### Pan-cancer expression landscape of HSP90AA1

To explore possible roles of HSP90AA1 in carcinogenesis, we analyzed its expression distribution of HSP90AA1 gene in 33 tumor tissues and normal tissues. HSP90AA1 was significantly upregulated in 27 cancer types, including ACC, BLCA, BRCA, CESC, CHOL, COAD, DLBC, ESCA, GBM, HNSC, KICH, KIRP, LGG, LIHC, LUAD, LUSC, OV, PAAD, PCPG, PRAD, READ, SKCM, STAD, TGCA, THCA, UCEC and UCS, and markedly downregulated in KIRC. Due to the lack of normal tissue data in the database, we only observed HSP90AA1 expression in LAML, MESO, and UVM. The data was download from TCGA, we obtained TCGA and Genotype-Tissue Expression (GTEx) data, as shown in **Figures 2M–P**. Next, we further validated the expression of HSP90AA1 in these 32 cancer types using CCLE database. As presented in **Figure 2Q**, the expression of HSP90AA1 in 32 malignant tumor tissues in CCLE database was consistent with that in TGCA database.

### Pan-cancer analysis of the correlation between HSP90AA1 expression and TNM classification

**Figure 3:**
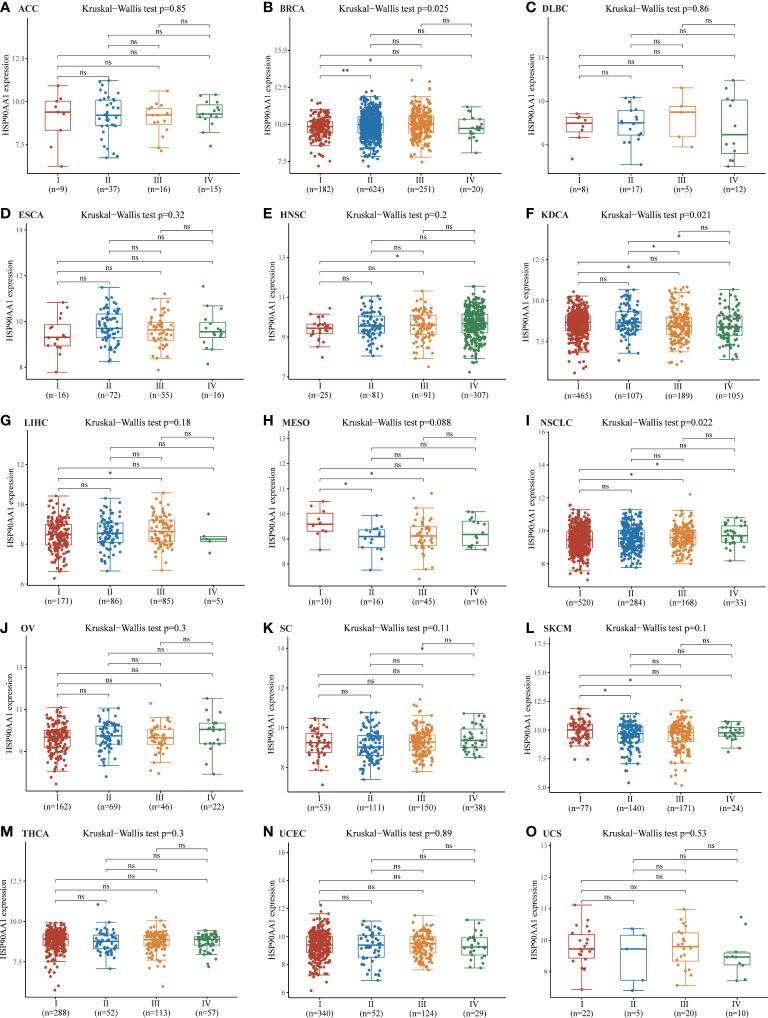
Correlations between the HSP90AA1 expression and the main pathological stages. ACC **(A)**, BRCA **(B)**, DLBC **(C)**, ESCA **(D)**, HNSC **(E)**, KDCA**(F)**, LIHC **(G)**, MESO **(H)**, NSCLC **(I)**, OV **(J)**, SC **(K)**, SKCM**(L)**, THCA **(M)**, UCEC **(N)**, UCS**(O)**. The analysis were investigated based on the TCGA data. Log2 (TPM+1) was used for log scale. *p < 0.05, **p < 0.01, and ns, no statistical significance. The statistical difference of stages was compared through Kruskal-Wallis test and the Mann-Whitney U test was used for comparison between stages.

Staging data were available for 15 tumors in the TGCA database, the upregulation of HSP90AA1 showed no significant difference in the expression of malignant tumors at different stages, including ACC, DLBC, ESCA, HNSC, LIHC, MESO, OV, SC, SKCM, THCA, UCEC and UCS (**Figure 3**). The expression of HSP90AA1 in different stages of BRCA showed that compared with stage I, the expression of HSP90AA1 in stage II and Stage III was significantly up-regulated, while the expression of HSP90AA1 in stage IV was slightly decreased (**Figure 3B**). Compared with stage I, the expression of HSP90AA1 in KDCA increased significantly in stage III, and was slightly down-regulated in stage IV, but the difference was not significant (**Figure 3F**). Compared with stage I, the expression of HSP90AA1 in NSCLC increased gradually with clinical stage, and the increase was significant in stage III and Stage IV, showing statistical difference (**Figure 3I**).

### The prognostic values of HSP90AA1 in pan-cancer

**Figure 4:**
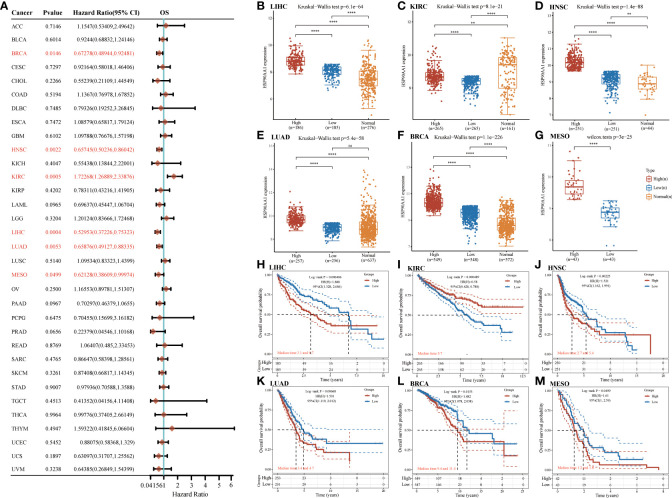
The overall survival (OS) analysis for HSP90AA1 in various human cancers determined by TCGA database. **(A)** The OS analysis for HSP90AA1 in various human cancers. **(B–G)** The expression of HSP90AA1 level in LIHC **(B)**, KIRC **(C)**, HNSC **(D)**, LUAD **(E)**, BRCA **(F)**, and MESO **(G)** compared with normal. **(H–M)** The OS plot of HSP90AA1 in LIHC **(H)**, KIRC **(I)**, HNSC **(J)**, LUAD **(K)**, BRCA **(L)**, and MESO **(M)**. **p < 0.01, ****p < 0.0001, asterisks (*) stand for significance levels. ns, no statistical significance.

Next, survival analysis for HSP90AA1 in human cancer was conducted. Four prognostic indices, consisting of OS, PFS, DFS and DSS, were included. As shown in **Figure 4A**, cox regression analysis showed that HSP90AA1 expression was significantly correlated with OS in 6 of the 32 cancers (marked in red), including LIHC, KIRC, HNSC, LUAD, BRCA and MESO (**Figures 4B**–**G**). Statistical analysis using Kruskal-Wallis method showed that HSP90AA1 expression in LIHC (**Figure 4B**), HNSC (**Figure 4D**), LUAD (**Figure 4E**), BRCA (**Figure 4F**) and MESO (**Figure 4G**) was significantly higher than normal tissues, while in KIRC (**Figure 4C**) was significantly lower than that in the control group. Kaplan-Meier survival curve was used to analyze the survival rates of patients with these six malignancies, and it was found that high expression of HSP90AA1 in LIHC (**Figure 4H**), HNSC (**Figure 4J**), LUAD (**Figure 3K**), BRCA (**Figure 4L**) and MESO (**Figure 4M**) was associated with poor survival rates, indicating unfavorable prognosis. But KIRC patients with higher expression of HSP90AA1 indicated better prognosis, as shown in **Figure 4I**.

**Figure 5:**
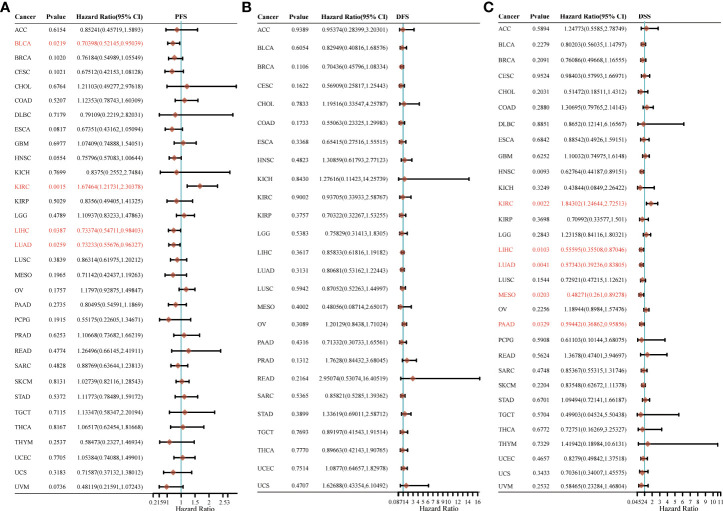
The progression free survival (PFS), **(A)** disease free survival (DFS) **(B)** and disease-specific survival (DSS) **(C)** analysis for HSP90AA1 in various human cancers determined by TCGA database.

For PFS, high expression of HSP90AA1 in BLCA, LIHC and LUAD had unfavorable prognosis but KIRC patients with higher expression of HSP90AA1 indicated better prognosis (**Figure 5A**). For DFS, no statistical significance of HSP90AA1 for predicting prognosis of patients in cancer types was observed (**Figure 5B**). For DSS, high expression of HSP90AA1 in BLCA, LIHC, LUAD, MESO and PAAD had unfavorable prognosis but KIRC patients with higher expression of HSP90AA1 indicated better prognosis (**Figure 5C**).

### Analysis of molecular mechanisms of HSP90AA1 regulation in LIHC and KIRC

**Figure 6:**
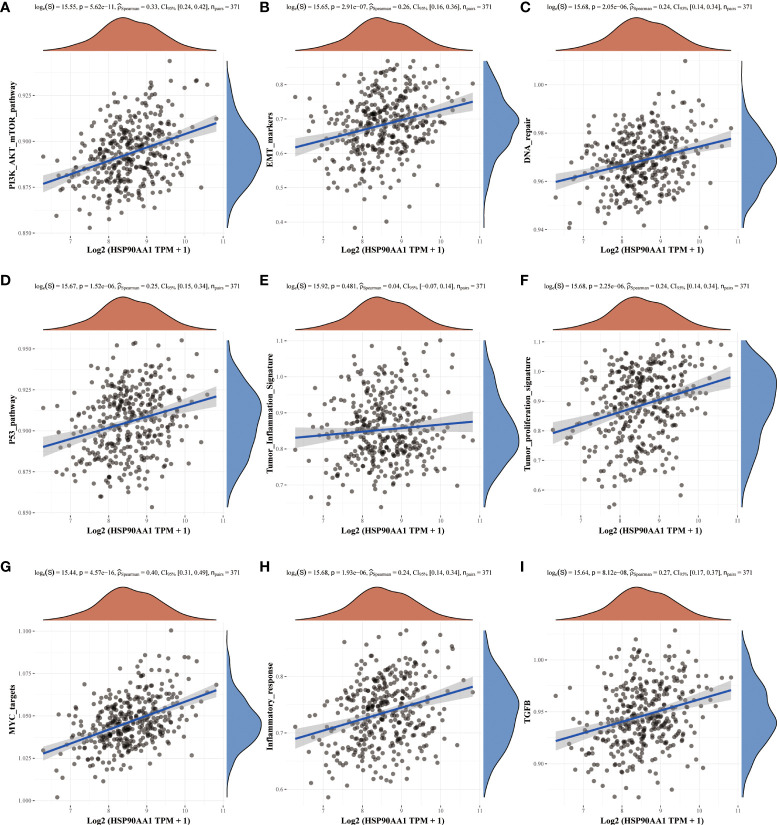
Correlation analysis between HSP90AA1 gene and pathway in LIHC. PI3K/AKT/mTOR pathway **(A)**, EMT markers **(B)**, DNA repair **(C)**, P53 pathway **(D)**, Tumor inflammation signature **(E)**, Tumor proliferation signature **(F)**, MYC targets **(G)**, Inflammatory response **(H)** and TGFB **(I)**, respectively. The upper orange density curve represents the trend in distribution of the gene expression, the blue density curve on the right represents the trend in distribution of pathway immune score.

**Figure 7:**
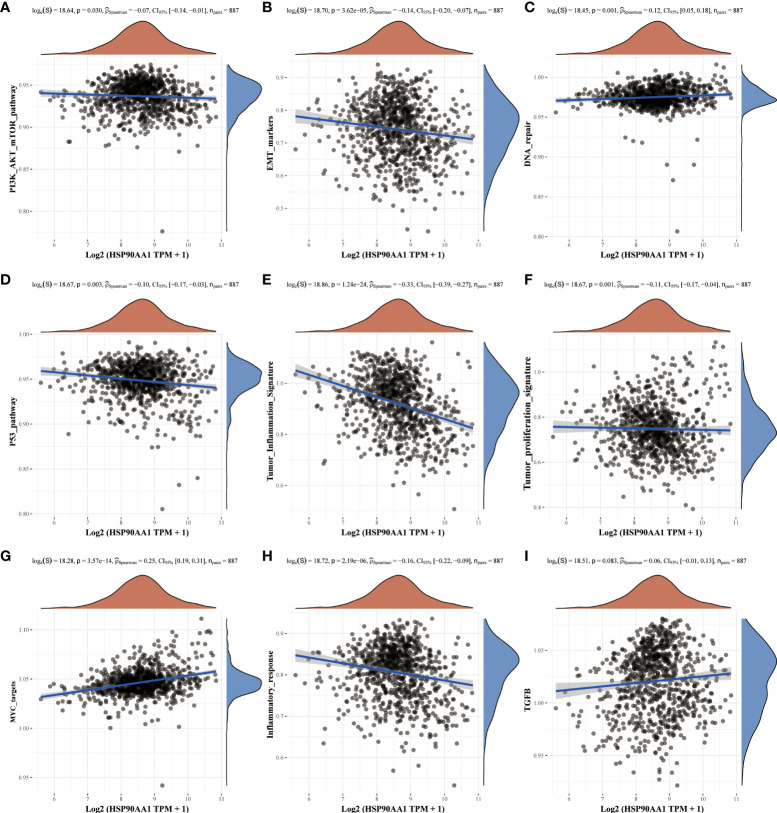
Correlation analysis between HSP90AA1 gene and pathway in KIRC. PI3K/AKT/mTOR pathway **(A)**, EMT markers **(B)**, DNA repair **(C)**, P53 pathway **(D)**, Tumor inflammation signature **(E)**, Tumor proliferation signature **(F)**, MYC targets **(G)**, Inflammatory response **(H)** and TGFB **(I)**, respectively. The upper orange density curve represents the trend in distribution of the gene expression, the blue density curve on the right represents the trend in distribution of pathway immune score.

Nine tumor mechanically-related pathways were included in this study. We found that the upregulation of HSP90AA in LIHC was correlated with all the included pathways, except tumor inflammation signaling pathways (**Figures 6A–I**). While in KIRC, expression of HSP90AA1 was negatively correlated with the EMT pathway, p53 pathway, tumor inflammatory signaling pathway and inflammatory response pathway, and positively correlated with MYC-targeting pathway, TGFB and DNA repair (**Figures 7A–I**).

### Pan-cancer analysis of the correlation between the HSP90AA1 expression and TMB, MSI

**Figure 8:**
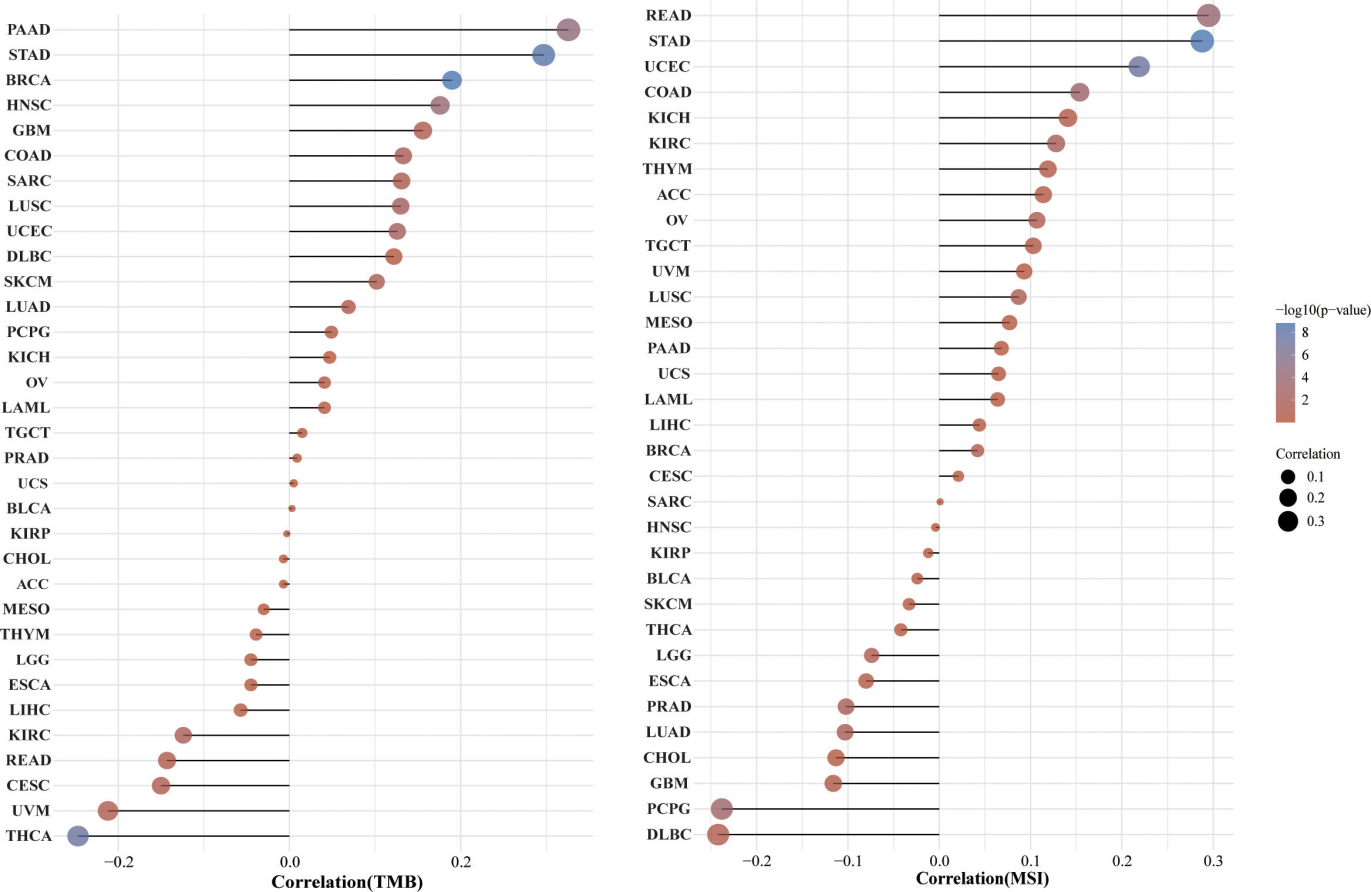
Correlation between the HSP90AA1 gene expression and TMB and MSI in pan-cancer. **(A)** The stick chart of the relationship between the HSP90AA1 gene expression and TMB in diverse tumors. The red curve represents the correlation coefficient, and the blue value represents the range. **(B)** The stick chart of the association between the HSP90AA1 gene expression and MSI in diverse tumors.

**Figure 9:**
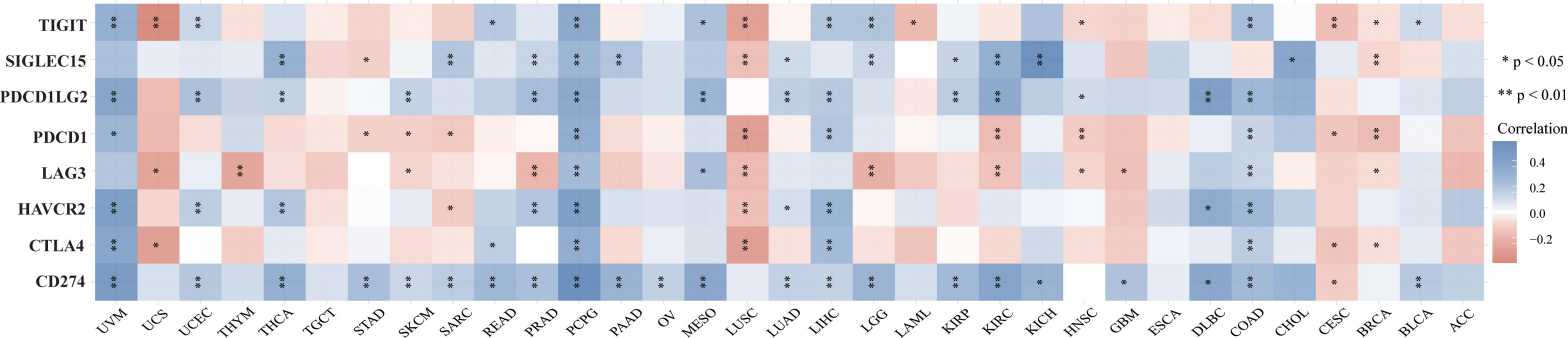
The heatmap of immune-checkpoint-related gene expression. The abscissa represents different tumor tissues, and the ordinate represents different immune-checkpoint-related gene. Each box in the figure represents the correlation analysis between the expression of the selected gene and the immune checkpoint in corresponding tumors. *p < 0.05, **p < 0.01, asterisks (*) stand for significance levels. Different colors represent the changes of correlation coefficients.

TMB and MSI are two emerging biomarkers associated with the immunotherapy response. HSP90AA1 expression level was significantly positively correlated with TMB in PAAD, STAD and BRCA, and significantly negatively correlated with TMB in KIRC, READ, CESC, UVM and THCA (**Figure 8A**). The correlation of the HSP90AA1 expression with MSI was also investigated, READ, UCEC and STAD exhibited positive correlations, and DLBC and PCPG exhibited negative correlations (**Figure 8B**). Immune monitoring affects the prognosis of tumor patients, and tumors use immune checkpoints such as PD-1, PD-L1 AND CTLA-4 to evade immune responses. We observed that up-regulation of HSP90AA1 in most tumors was positively correlated with PDCD1LG2 and CD274 immune checkpoint genes, with statistical differences. In UVM, PCPG, LIHC and COAD, HSP90AA1 was positively correlated with most of the immune checkpoint genes, especially PCPG, which was significantly positively correlated with all the immune checkpoint genes. While in UCS, LUSC and CESC, the upregulation of HSP90AA1 was negatively correlated with most of the immune checkpoint genes, especially LUSC (**Figure 9**).

### Correlation between HSP90AA1 and immune cells in pan-cancer

**Figure 10:**
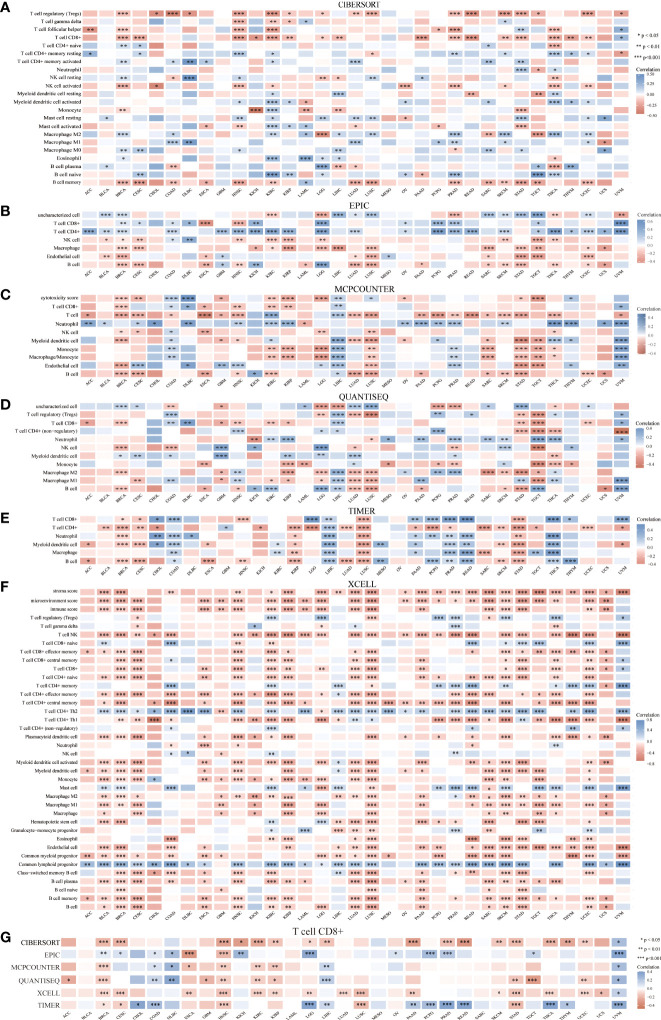
Correlation between HSP90AA1 and immune cells in multiple cancers. **(A)** CIBERSORT, **(B)** EPIC, **(C)** MCPCOUNTER, **(D)** QUANTISEQ, **(E)** TIMER, **(F)** XCELL, **(G)** Correlation between HSP90AA1 and CD8+ T Cell in multiple cancers. *p < 0.05,**p < 0.01,***p < 0.001, asterisks(*)stand for signifcance levels.Different colors represent the changes of correlation coeffcients.

Given that HSP90α upregulation has been correlated with cancer, it is important to understand its unique functions. Thus, the correlation of HSP90AA1 expression with immune cell was evaluated. We determined the expression correlation of HSP90AA1 with immune cells in multiple cancers using six databases. As presented in **Figures 10A–F**. we found that HSP90AA1 expression was significantly positively associated with CD8+ T cells in COAD, DLBC and UVM, and negatively associated with CD8+ T cells in ESCA, GBM, HNSC, KIRC, KIRP, UCEC and STAD (**Figure 10G**).

### Analysis of HSP90α for diagnosis malignant tumors

**Figure 11:**
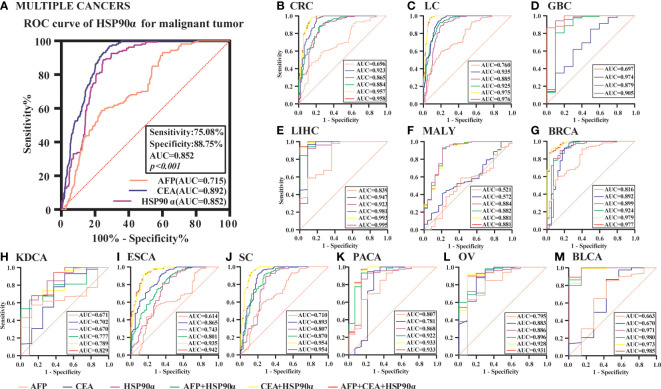
The ROC curve analyzed the diagnosis efficiency of HSP90α in malignant tumors. **(A)** The ROC curve analyzed the diagnosis efficiency of HSP90α, CEA and AFP for total malignant tumors. **(B–M)** The ROC curve analyzed the diagnosis efficiency of HSP90α, CEA, AFP, AFP+HSP90α, CEA+HSP90α and AFP+CEA+HSP90α for each malignant tumor.

ROC curve analysis was performed to determine the cut-off value of HSP90α malignant tumor predictions. The results revealed that the AUC of HSP90α was 0.852 and the optimal clinical cut-off level was 50.34 ng/mL, which provided a 75.08% sensitivity and an 88.75% specificity (*p* < 0.001). The AUC of CEA was 0.892, (sensitivity = 71.34%, specificity = 96.87%, *p* < 0.001), the AUC of AFP was 0.715, (sensitivity = 76.06%, specificity = 58.75%, *p* < 0.001). The results suggested HSP90α has higher sensitivity than CEA and higher specificity than AFP. (**Figure 11A**). As shown in **Table 3**, plasma HSP90α levels were positively correlated with the serum levels of CEA (r = 0.171; *p* < 0.001), not AFP (r = 0.068; *p* = 0.092). **Figures 11B–M** shows the AUC values of independent diagnosis and combined diagnosis, which are HSP90α, CEA, AFP, HSP90α&AFP, HSP90α&CEA and HSP90α&CEA&AFP, respectively. The AUC, sensitivity, and specificity of HSP90α&CEA group in the CRC, LC, GBC, MALY, BRCA, KDCA, ESCA, SC, PACA, and OV cancers are generally higher than that of the HSP90α &AFP group.

**Table 3:** Correlation between HSP90α and other biomarkers in malignant tumors.

Index	R (with HSP90α)	95% CL	*p*-value
CEA	0.171	0.093-0.247	<0.001
AFP	0.068	-0.011-0.146	0.092

Carcinoma Embryonic Antigen (CEA), Alpha-Fetoprotein (AFP).

R > 0.1 and p-value < 0.05 were set as selection criteria for identifying as statistically significant.

For BLCA, the combined diagnosis of HSP90α+CEA+AFP has the largest AUC, but there is no significant difference between HSP90α+CEA and HSP90α+CEA+AFP in sensitivity and specificity, the both AUC are 1, while the sensitivity and specificity of both groups are 100%. The AUC of independent diagnosis HSP90α and CEA in BLCA is less sensitive and specific than the combined diagnosis of HSP90α+AFP. For LIHC, the combined diagnosis of HSP90α+CEA+ AFP has the largest AUC, while there is no significant difference between HSP90α+AFP and HSP90α+CEA+AFP in sensitivity and specificity; the AUC of HSP90α+CEA was higher than HSP90α+AFP, while there was no difference in sensitivity and specificity. Most AUC of HSP90α were between 0.6 and 1.0 in different tumor groups, indicating its certain diagnostic value in various types of malignant tumors (AUC > 0.6, *p* < 0.05). The optimal clinical cut-off level, sensitivity and specificity was shown in **Table 4**.

**Table 4:** Main parameters of ROC curve analysis results in tumor groups.

Variables	95%Cl	Sensitivity(%)	Specificity(%)	Cut-off	*p*-value
CRC					
AFP	0.635-0.752	74.19	58.75	2.53	<0.001
CEA	0.886-0.955	79.57	97.50	3.34	<0.001
HSP90α	0.817-0.905	76.34	88.75	50.34	<0.001
AFP+HSP90α	0.840-0.928	68.82	92.50		<0.001
CEA+HSP90α	0.929-0.985	86.02	98.12		<0.001
AFP+CEA+HSP90α	0.930-0.986	86.02	98.12		<0.001
LC					
AFP	0.714-0.802	79.74	60.00	2.63	<0.001
CEA	0.905-0.957	83.70	93.12	2.69	<0.001
HSP90α	0.849-0.915	80.62	88.75	50.34	<0.001
AFP+HSP90α	0.900-0.951	81.94	91.37		<0.001
CEA+HSP90α	0.961-0.990	91.63	98.43		<0.001
AFP+CEA+HSP90α	0.962-0.990	91.63	98.43		<0.001
GBC					
AFP	0.622-0.765	50.00	85.00	5.48	0.045
CEA	0.938-0.992	100.00	86.25	2.15	<0.001
HSP90α	0.820-0.924	90.00	80.62	47.00	<0.001
AFP+HSP90α	0.751-1.000	60.00	100.00		<0.001
CEA+HSP90α	1.000	100.00	100.00		<0.001
AFP+CEA+HSP90α	1.000	100.00	100.00		<0.001
LIHC					
AFP	0.775-0.891	63.64	98.12	8.52	<0.001
CEA	0.903-0.976	90.91	96.87	3.13	<0.001
HSP90α	0.872-0.958	90.91	92.50	53.28	<0.001
AFP+HSP90α	0.952-1.000	81.82	100.00		<0.001
CEA+HSP90α	0.980-1.000	81.82	100.00		<0.001
AFP+CEA+HSP90α	0.984-1.000	90.91	100.00		<0.001
MALY					
AFP	0.446-0.595	58.33	58.75	2.53	0.754
CEA	0.497-0.645	79.17	41.25	1.03	0.243
HSP90α	0.829-0.927	79.17	93.12	55.60	<0.001
AFP+HSP90α	0.793-0.971	50.00	98.75		<0.001
CEA+HSP90α	0.792-0.971	54.17	99.37		<0.001
AFP+CEA+HSP90α	0.792-0.970	54.17	99.37		<0.001
BRCA					
AFP	0.755-0.867	60.00	91.25	6.35	<0.001
CEA	0.841-0.932	75.00	98.12	3.89	<0.001
HSP90α	0.849-0.937	85.00	89.37	51.63	<0.001
AFP+HSP90α	0.866-0.982	70.00	98.75		<0.001
CEA+HSP90α	0.959-0.998	77.50	98.12		<0.001
AFP+CEA+HSP90α	0.956-0.998	77.50	98.75		<0.001
KDCA					
AFP	0.595-0.742	87.50	57.50	2.49	0.009
CEA	0.627-0.770	50.00	93.12	2.69	0.09
HSP90α	0.593-0.740	75.00	62.50	41.45	0.163
AFP+HSP90α	0.654-0.899	12.50	100.00		<0.001
CEA+HSP90α	0.625-0.953	25.00	100.00		<0.001
AFP+CEA+HSP90α	0.665-0.993	25.00	100.00		<0.001
ESCA					
AFP	0.548-0.676	71.43	55.00	2.37	0.006
CEA	0.814-0.906	66.23	90.62	2.46	<0.001
HSP90α	0.682-0.797	63.64	80.62	47.00	<0.001
AFP+HSP90α	0.739-0.862	51.95	92.50		<0.001
CEA+HSP90α	0.900-0.971	76.62	95.62		<0.001
AFP+CEA+HSP90α	0.911-0.973	76.62	95.62		<0.001
SC					
AFP	0.650-0.765	41.94	93.12	6.53	<0.001
CEA	0.848-0.928	79.57	91.87	2.52	<0.001
HSP90α	0.753-0.854	70.97	89.37	51.63	<0.001
AFP+HSP90α	0.819-0.920	68.82	92.50		<0.001
CEA+HSP90α	0.926-0.983	82.80	96.87		<0.001
AFP+CEA+HSP90α	0.925-0.983	82.80	96.87		<0.001
PACA					
AFP	0.740-0.863	57.14	93.12	6.53	<0.001
CEA	0.712-0.840	71.43	95.62	2.98	0.007
HSP90α	0.809-0.915	85.71	86.25	49.33	<0.001
AFP+HSP90α	0.833-1.000	50.00	99.37		<0.001
CEA+HSP90α	0.835-1.000	71.43	100.00		<0.001
AFP+CEA+HSP90α	0.832-1.000	71.43	100.00		<0.001
OVC					
AFP	0.727-0.853	70.00	85.00	5.48	0.003
CEA	0.825-0.928	80.00	88.12	2.32	<0.001
HSP90α	0.829-0.930	80.00	89.37	51.63	<0.001
AFP+HSP90α	0.798-0.995	50.00	100.00		<0.001
CEA+HSP90α	0.838-1.000	60.00	100.00		<0.001
AFP+CEA+HSP90α	0.846-1.000	60.00	100.00		<0.001
BLCA					
AFP	0.586-0.735	57.14	85.00	1.46	0.253
CEA	0.593-0.740	57.14	86.87	2.21	0.242
HSP90α	0.933-0.991	85.71	100.00	79.10	<0.001
AFP+HSP90α	0.943-1.000	85.71	100.00		<0.001
CEA+HSP90α	0.927-1.000	85.71	99.37		<0.001
AFP+CEA+HSP90α	0.956-1.000	85.71	100.00		<0.001

Carcinoma Embryonic Antigen (CEA), Alpha-Fetoprotein (AFP), Heat shock protein 90α (HSP90α).

## Discussion

The high incidence and mortality of cancer pose a serious threat to human health (1, 3). The most common cancer treatments include surgical excision, radiation and adjuvant chemotherapy, but their effectiveness remains limited. Early detection and effective treatment are important conditions to improve the prognosis of cancer patients. Pan-cancer analysis can reveal similarities and differences between different cancers, providing insights into cancer prevention and the design of personalized treatment strategies (27). In recent years, more and more studies have focused on genome-wide pan-cancer analysis, revealing gene mutations, RNA changes and cancer driver genes related to cancer genesis and development, which is of great significance for the early diagnosis of cancer and the identification of sensitive biomarkers (4, 28). This study analyzed the expression of HSP90α and its coding gene HSP90AA1 in different tumors from the plasma level of clinical patients, tissue level and gene level of pan-cancer. Analysis of HSPP90A1 expression in 32 tumors in TCGA and CCLE databases showed that HSP90AA1 was significantly up-regulated in most tumors compared with normal tissues, but down-regulated in a few tumors, such as KIRC. The expression of HSP90AA1 in a variety of tumors was found to be correlated with tumor prognosis, immune cell invasion, immune checkpoint genes and a variety of signaling pathways, but the correlation was inconsistent among different tumors. HSP90AA1 expression was strongly correlated with immunomodulatory biomarkers in some tumors. We also found that, different from gene expression level, HSP90α expression in plasma of many tumor patients was higher than that of normal subjects, but its concentration gradient was different. The expression of HSP90α was up-regulated resulting the prognosis was worse, but the opposite is true for individual tumors, such as KIRC. However, it is confirmed that in many tumors, they can be used as biomarkers for tumor diagnosis and prognostic analysis, and the combined diagnosis of CEA or AFP is better.

HSP90α is an ATP-dependent molecular chaperone that regulates late maturation, activation, and stability of various client proteins (30). HSP90α protein plays an important role in important cellular processes and regulatory pathways, such as apoptosis, cell cycle control, cell signaling, cell viability, protein folding and degradation, when interacting with client proteins and co-partners (31). Associations have been observed between HSP90α overexpression and disease conditions, such as links between various types of cancer, viral infections, inflammation and neurodegenerative diseases, suggesting that HSP90α may contribute to cancer progression (32–34). Many HSP90α client proteins are involved in signal transduction and other important pathways that are particularly important in malignant tumors (35). An interesting phenomenon was found in the analysis of pan-cancer and comparative analysis of serological data in this study. The expression of HSP90α in plasma was significantly upregulated in most tumors, but slightly increased in kidney related tumors, showing no difference compared with normal patients. Similarly, an immuno-histochemical clinical study of 109 patients with KIRC found a slight increase in HSP90 expression in tissues (36). The opposite effect was observed at the genetic level, the expression of HSP90AA1 in KIRC was lower than that in normal tissues, and the difference was statistically significant. We found that expression of HSP90AA1 in KIRC was negatively correlated with the EMT, p53, tumor inflammatory signaling pathway and inflammatory response pathway, and positively correlated with MYC-targeting, TGFB and DNA repair. It has been found that client proteins of HSP90, including HIF-1α and receptor tyrosine kinases MET and KIT, are elevated in KIRC, while KIT is overexpressed and its downstream targets, AKT and RAF, are also HSP90 client proteins. Predictably, HSP90α, as a molecular chaperone, can regulate the expression of multiple tumorigenesis proteins in KIRC, this requires further research.

In tumors, the proliferation of tumor cells seems to have been in a “stress” state, which makes it difficult to maintain the balance within the protein (37), and canceration increases the dependence of tumor cells on HSP90α (38). HSP90α overexpression by maintaining the stability of oncoproteins promotes survival, proliferation, new blood vessel formation and metastasis, and indirectly regulates DNA damage and cell metabolism (39). Studies also found that these tumor cell lines can autocrine HSP90α to promote tumor migration, invasion and metastasis, including SH-76 hybridoma cells, HT-1080 fibro sarcoma cells, MDA-MB-231 breast cancer cells, MCF-7 breast cancer cells, HCT-8 colorectal cancer cells, Bladder cancer cells, B16 melanoma cells, PC3 prostate cancer cells, CaoV-3 ovarian cancer cells and HepG2 liver cancer cells (40, 41). Chen et al. study showed that pancreatic cancer infiltrated macrophages was also secreted a large amount of HSP90α (37). Some studies have also found that HSP90α participated in the growth and development of tumors on the cell surface, and the content of HSP90α is not only related to age, tumor volume, staging and metastasis or not, but also reflected in preoperative and postoperative, disease development and other aspects (14). In contrast to KIRC, the expression of HSP90AA1 was significantly up-regulated in LIHC, and its coding protein HSP90α was also significantly up-regulated in tissue and plasma. Further research showed that upregulation of HSP90AA1 in LIHC was correlated with all the included pathways. It has been found that HSP90 can bind to Pyruvate kinase M2 (PKM2) and subsequently increase the abundance of PKM2 in LIHC cells. By mechanism, HSP90 increases PKM2 phosphorylation at THR-328, and protein kinase glycogen synthase kinase-3 β (GSK-3β) forms a protein complex with HSP90 and PKM2, and directly mediates HSP90-induced THR-328 phosphorylation of PKM2 (42).

Transcription of HSP90AA1 is regulated by a variety of transcription complexes. In breast cancer cells, the growth hormone prolactin induces HSP90AA1 expression through STAT5 (43). Nf-kb or RELA can also induce HSP90AA1 expression, which may explain the pro-survival ability of NF-KB-driven transcription (44). Conversely, tumor suppressor STAT1 was found to inhibit stress-induced HSP90AA1 expression (45). HSP90α as a cancer enabler is considered to be an essential factor for malignant transformation and progression. It is not consistent with the research results of Zuehlke et al. (46), compared with the expression of HSP90α at protein and plasma level, the expression of HSP90AA1 gene level increased slightly in the pan cancer analysis. One of the possible reasons is that whole-genome sequencing of all tumor types and cancer cell lines revealed 115 different mutations in HSP90AA1 open reading frame, which may lead to the failure of HSPAA1 transcription in tumors and reduce the degree of malignancy of tumors (46). In addition, HSP90α can be secreted extracellular through the exosome pathway, resulting in increased expression in tissues and body fluids. It has been found that HSP90α can increase the movement of tumor cells to increase metastasis and invasion (47).

Overall, the higher the expression of HSP90α is in most malignant tumors, the lower the overall survival rate is, which is consistent with most clinical studies on single tumors (18). In a study of the diagnostic and prognostic value of HSP90α in gastric cancer, HSP90α of plasma was not associated with survival in patients with gastric cancer (48). While HSP90α was identified as an independent prognostic factor for nasopharyngeal carcinoma by univariate and multivariate Cox proportional risk regression analysis in a clinical study (49). A retrospective study measured HSP90α levels before and after treatment in 231 patients with lung cancer and found that HSP90α levels were associated with lung cancer treatment response and patient outcomes (50). In addition, studies have shown that for early chemotherapy of advanced NSCLC, a significant decrease in plasma HSP90a value is an important predictor of chemotherapy effectiveness (51). It has also been found that HSP90α is associated with the prognosis of human esophageal squamous cell carcinoma, and may be involved in the regulation of cyclin B1 expression (52). The prognostic values analysis of HSP90α was showed in human cancer, the OS analysis of HSP90α has statistically significant only in six types of cancers. The high expression of HSP90α in LIHC, HNSC, LUAD, BRCA and MESO had unfavorable prognosis, but KIRC patients with higher expression of HSP90α indicated better prognosis. It can be seen that HSP90α has predictive value for the prognosis of most tumors, but not all tumors from the analysis of pan-cancer and single-tumor studies. Due to the specificity of HSP90α, its changes before and after treatment in many tumors are more valuable for predicting the prognosis after treatment.

Numerous studies have confirmed that tumor immune cell infiltration could influence the efficacies of chemotherapy, radiotherapy, or immunotherapy and prognosis of cancer patients (53–55). Studies have measured the concentration of Hsp90α and its antibody in the serum of patients with psoriasis, and compared with healthy individuals, found that the immune response of patients with psoriasis increased, suggesting that inhibition of Hsp90α may represent a new treatment method for psoriasis (56). In the study of fin regeneration of zebrafish, it was found that the immune system and related pathways JAK2α and STAT1b are involved in regeneration, and HSP90α plays an important role in the initiation and promotion of regeneration (57). Recent studies suggest that extracellular HSP90α induces MyD88-IRAK complex-associated IKKα/β-NF-κB/IRF3 and JAK2/TYK2-STAT-3 signaling pathways in macrophages to promote tumor M2 polarization (58). In a study of pancreatic neoplasms, we found that myeloid macrophages and their secretion of HSP90α induce the development of pancreatic ductal adenocarcinoma and promote epithelial mesenchymal transformation, migration, and invasion. It was found that macrophages not only secreted HSP90α themselves, but also secreted interleukin-6 and interleukin-8 can induce JAK2-STAT3 signal transduction in pancreatic ductal cell carcinoma cells, and stimulate the secretion and expression of HSP90α (16). Our work suggested that HSP90AA1 was significantly positively or negatively correlated with various immune cells in the six latest algorithms, including CD8+ T cell, macrophage, B cell, neutrophil, NK cell, Myeloid dendritic cell, Monocyte and T cell regulatory et al. in malignant tumor. We found that CD8+ T cell was positively correlated with HSP90AA1 in COAD, DLBC and UVM, and negatively correlated with HSP90AA1 in ESCA, GBM, HNSC, KIRC, KIRP, UCEC and STAD in the six latest algorithms. CD8+ T cells was significantly elevated in tumors and infections, and elevated in tumors often indicate poor prognosis. In the prognostic analysis of OS, we found that the increase of HSP90AA1 in KIRC indicates a better prognosis, which is consistent with the negative correlation of HSP90AA1 in immune cell CD8+ T analysis on the prognosis of KIRC. This suggested that the elevation of HSP90α also may be used as a clinical prognostic biomarker. Other immune cell such as Macrophage and B cell were correlated with various cancer in the five latest algorithms, neutrophil, NK cell, Myeloid dendritic cell, Monocyte and T cell regulatory were correlated with various cancer in the four latest algorithms. This will require much more research to segment.

Cancer immunotherapy blocks multiple immune checkpoints, which can significantly target tumor therapy and significantly improve the therapeutic effect of cancer. Through cyto-chemical screening of approximately 200,000 compounds, it has been found that HSP90 inhibitors reduce pD-L1 surface expression through a mechanism that appears to be involved in the regulation of major transcriptional regulators, namely STAT-3 and C-MYC. Meanwhile, HSP90 inhibitors can also regulate the surface expression of the extra checkpoint protein, namely PD-L2 (59). Knowing that HSP90 overexpression may play a role in the development of cancer, researchers are investigating the use of HSP90 inhibitors to inhibit HSP90 overexpression and thus treat cancer. Drug screening and functional assays revealed that HSP90 controls protein stability of the nuclear transcription factor STAT1 in a variety of different cancer cells, thereby promoting subsequent gene expression of immune checkpoint molecules (IDO1 and PD-L1). The study found that targeting HSP90 enhanced the efficacy of PD-1 blockade therapy in different mouse models of pancreatic cancer (60). The sensitivity of tumors to immunotherapy is inconsistent. Pan-cancer analysis of this study provides the sensitivity of most tumors in the database to immunomodulatory biomarkers and helps researchers selecting appropriate treatment strategies.

The content of HSP90α is very low in normal human plasma, it can be actively secreted extracellular by the cell to play a physiological and pathological processes role when stress or malignancy occurred. When tumors occurred, they can be specifically secreted out of tumor cells and entered into the blood, then the levels of HSP90α are significantly increased in blood (61, 62). The plasma level of HSP90α in patients with malignant melanoma was significantly higher than that in healthy subjects, and the AUCs value was higher than lactate dehydrogenase (LDH), with higher sensitivity (76.7%) and predictive value (78.8%) (63). Another study compared plasma HSP90α value in 976 patients with gastric cancer and found that HSP90α value was significantly higher than that in healthy people, and it had moderate diagnostic performance (48). In a clinical study of patients with hepatocellular carcinoma, plasma HSP90α has been found to be of diagnostic value for early hepatocellular carcinoma, and the combination of AFP can improve the diagnostic efficiency (22). We can also see the diagnostic value found in other tumor studies, such as colorectal cancer (21), nasopharyngeal carcinoma (49), lung cancer (64). In this study, plasma HSP90α in the malignant tumor group was significantly higher than that in the normal group (P < 0.001). The plasma HSP90α levels in all tumor groups were generally elevated, except for KDCA (P < 0.05). The increase in LIHC group was the most significant, higher than that in other tumor groups (P < 0.001). There was no significant difference among other groups (P > 0.05). The area under the curve (AUC) of plasma HSP90α is concentrated between 0.6 and 1.0 in this clinical group, which indicates that it can be used as a potential diagnostic biomarker in most tumors and has been confirmed in many single-tumor studies. The diagnostic efficacy of HSP90α combined with CEA and/or AFP in various tumors has been significantly improved, indicating that it can be used as a biomarker in clinical early screening diagnosis.

There are some limitations of this study. First of all, the clinical data in this study were collected from a single diagnosis and treatment center. In addition, this study is a retrospective study. Because the author is interested in the diagnosis, prognosis and immunotherapy of HSP90α in diffuse cancer analysis, the expression level of plasma HSP90α tumor patients in our institution was only reviewed. Future multi-cancer center studies and prospective studies of HSP90α should be established. Extensive cancer analysis provides data support for prospective, big data studies.

## Conclusions

High expression of HSP90α is significantly associated with poor prognosis in several cancers. The upregulation of HSP90AA1 was strongly correlated with many immune cell infiltrates. There was a strong correlation between HSP90AA1 expression and immune checkpoint gene. Pan-cancer analysis and clinical data analysis provided a better understanding of the differences in HSP90AA1 gene and HSP90α protein expression in plasma and tissues of different cancers.

## Data availability statement

Publicly available datasets were analyzed in this study. This data can be found here: https://genome-cancer.ucsc.edu/ and https://portal.gdc.cancer.gov/.

## Ethics statement

This study was approved by the Ethics Committee of Shaanxi Provincial Cancer Hospital (No. 2021089).

## Author contributions

ZY drafted the manuscript and carried out statistical analysis. CC conceived and designed the study. ZY acquired the data and contributed to the interpretation of the data. LW is responsible for data quality control. All authors contributed to the article and approved the submitted version.

## Conflict of interest

The authors declare that the research was conducted in the absence of any commercial or financial relationships that could be construed as a potential conflict of interest.

## Publisher’s note

All claims expressed in this article are solely those of the authors and do not necessarily represent those of their affiliated organizations, or those of the publisher, the editors and the reviewers. Any product that may be evaluated in this article, or claim that may be made by its manufacturer, is not guaranteed or endorsed by the publisher.
